# Systematic review of school tobacco prevention programs in African countries from 2000 to 2016

**DOI:** 10.1371/journal.pone.0192489

**Published:** 2018-02-06

**Authors:** Akihiro Nishio, Junko Saito, Sachi Tomokawa, Jun Kobayashi, Yuka Makino, Takeshi Akiyama, Kimihiro Miyake, Mayumi Yamamoto

**Affiliations:** 1 Health Administration Center, Gifu University, Gifu, Japan; 2 Japanese Consortium for Global School Health Research, Nishihara, Japan; 3 School of Public Health, The University of Tokyo, Tokyo, Japan; 4 Faculty of Education, Shinshu University, Nagano, Japan; 5 Faculty of Medicine, University of the Ryukyus, Nishihara, Japan; 6 Nagano College of Nursing, Komagane, Japan; TNO, NETHERLANDS

## Abstract

**Background:**

The World Bank has reported that global smoking rates declined from 2000 to 2012, with the only exception found in males in Sub-Saharan Africa. Sub-Saharan Africa is considered to be in stage one of the tobacco epidemic continuum. To address this problem, school-based programs for smoking prevention are considered cost-effective and promising. Since tobacco prevention programs are influenced by social competence or customs of each country, tobacco prevention programs that have success in Western countries are not always effective in African countries. Therefore, the current study systematically reviewed relevant literature to examine the effects of these types of programs in African countries.

**Method:**

Online bibliographic databases and a hand search were used. We included the studies that examined the impact of school-based programs on preventing tobacco use in Africa from 2000 to 2016.

**Results:**

Six articles were selected. Four were conducted in South Africa and two were performed in Nigeria. Four programs were systematically incorporated into annual curriculums, targeting 8^th^ to 9^th^ graders, while the other two were temporary programs. All programs were based on the hypothesis that providing knowledge and/or social skills against smoking would be helpful. All studies utilized smoking or polydrug use rates to compare outcomes before/after intervention. There were no significant differences between intervention and control groups in three studies, with the other three demonstrating only partial effectiveness. Additionally, three studies also examined change of knowledge/attitudes towards smoking as an outcome. Two of these showed significant differences between groups.

**Conclusion:**

All RCTs studies showed no significant change of smoking-rate by the intervention. The effectiveness of intervention was observed only in some sub-group. The cohort studies showed school-based interventions may be effective in improving knowledge and attitudes about smoking. However, they reported no significant change of smoking-rate by the intervention.

## Background

Smoking is one of the largest threats to public health. Smoking is associated with nearly one out of every three deaths from cancer, nearly one out of every five deaths from heart disease, and importantly, nine out of ten lung cancer deaths [1,2]. In high-income countries, the trend of smoking rates is in decline [3]. Therefore, it is estimated more than 80 percent of the world’s smoking-related deaths will be in low- and middle-income countries by the year 2030 [4,5]. The World Bank reported that smoking rates were seen to decline from 2000 to 2012 in almost all regions of the world, such as the Euro area, East Asia/Pacific, Latin America/Caribbean, North America, and South Asia in both males and females [6]. The only exception noted were males in Sub-Saharan Africa. The estimated prevalence of smoking in sub-Saharan Africa in 2012 was 22% in males (increasing from the level of 21% in 2000) and 3% in females. Thus, Sub-Saharan Africa might still be in stage one of the tobacco epidemic continuum [7].

Adolescence is one of the highest risk developmental phases for the initiation of tobacco use. In Africa, the estimated prevalence of smoking is 9.3% for boys, 3.8% for girls, and 6.6% for both sexes [8]. This developmental period also avails itself as a critical chance to prevent tobacco use. Therefore, intervention programs in schools have the unique advantage of reaching a large number of at-risk youth within a short period of time, making these programs one of the most promising methods to prevent tobacco use. In 2000, UNESCO, UNICEF, and The World Bank developed the Focusing Resources on Effective School Health (FRESH) initiative to strengthen health promotion and educational activities in schools [9]. FRESH consists of four components: 1) health-related school policies; 2) water, sanitation, and the environment; 3) skills-based health education; and 4) health and nutrition Services. Further, FRESH recommends that governments implement school-based health programs, including tobacco prevention, in efficient, realistic, and result-oriented ways [9]. Taking this information into account, tobacco prevention school programs should be implemented with the best method based on scientific evidence throughout Africa.

The results of previous studies on the effectiveness of school-based programs in preventing tobacco use among school children are mixed. According to a review by Thomas et al. reported longest follow-up found an overall significant effect with average 12% reducation in starting smoking compared with controls, but no effect for all trials pooled at less than 1 year. However, trials of combined social competence/social influence curricula had a significant effect on smoking prevention at both follow-up periods[10]. While a review of 25 studies by Skara and Sussman provided long-term empirical evidence of the effectiveness in preventing or reducing substance use for up to 15 years after program completion [11], little to no rigorous evidence of effectiveness was found in a review of eight studies in individuals up to 18 years old (i.e., 12^th^ grade) [12]. However, almost all of the studies selected in these reviews contain data from high-income countries. Tobacco prevention programs demonstrated to perform best in developed countries will not always perform well in African countries, because tobacco prevention programs require much social information to create curricula. To the best of our knowledge, no systematic reviews have been performed to quantify the effects of school-based programs on preventing tobacco use among school children in Africa, where the tobacco epidemic is still occurring. Therefore, to provide contemporary school-based prevention programs in African countries, following establishment of the FRESH framework, the current manuscript conducted a systematic review of all available research on this subject.

## Material and methods

The protocol for this systematic review was registered in February 20, 2017, in the PROSPERO database: https://www.crd.york.ac.uk/PROSPERO/display_record.asp?ID=CRD42017056711

### Search strategy for relevant articles

In October 2016, PubMed, Web of Science, SCOPUS, ERIC, PsycINFO, Popline, CINAHL, and CENTAL were used to search for relevant articles. We created the search terms based on a previous review, and then adapted them to all other databases according to vocabulary and syntax of each database.

We adapted the search formula to each database style. The basic formula utilized was as follows:

school* AND (child* OR adolescen* OR student* OR pupil*) AND (Africa OR Cameroon OR "Central African Republic" OR Chad OR Congo OR "Democratic Republic of the Congo" OR "Equatorial Guinea" OR Gabon OR Burundi OR Djibouti OR Eritrea OR Ethiopia OR Kenya OR Rwanda OR Somalia OR “South Sudan” OR Sudan OR Tanzania OR Uganda OR Angola OR Botswana OR Lesotho OR Malawi OR Mozambique OR Namibia OR "South Africa" OR Swaziland OR Zambia OR Zimbabwe OR Benin OR "Burkina Faso" OR "Cape Verde" OR "Cote d'Ivoire" OR Gambia OR Ghana OR Guinea OR "Guinea-Bissau" OR Liberia OR Mali OR Mauritania OR Niger OR Nigeria OR Senegal OR "Sierra Leone" OR Togo OR Algeria OR Egypt OR Libya OR Morocco OR Tunisia) AND (tobacco OR smok*)

In addition, we hand-searched five key journals, including School Health Research, Health Education Research, Health Promotion International, Tropical Medicine & International Health, and Cochrane Library.

Two reviewers (AN and MY) independently searched articles, utilizing identical methodology, and evaluated articles to find relevant studies following pre-established inclusion criteria. Any disagreements between the reviewers were resolved by discussion and, if necessary, by consulting a third reviewer (ST).

### Inclusion criteria

The title and abstracts of the results generated from the searching database were screened using the following inclusion criteria:

1. Study design: all quantitative study designs, including randomized controlled trials (RCT), non-randomized trials, cohort studies (controlled and uncontrolled), and cross-sectional studies.
2. Study objectives: studies that examined the impact of school-based programs on preventing tobacco use.
3. Participants: students in Grades 1 to 12
4. Location: African counties
5. Program: any type of school-based program targeting students to prevent smoking
6. Publishing: studies published in a peer-reviewed journal and written in English from January 1, 2000 to June 30, 2016

We examined each of the selected articles from the viewpoint of country, targeted children, and type of intervention. We also evaluated the risk of bias of RCTs or cluster-randomized control trials by the Cochrane Collaboration’s tool for assessing risk of bias [13]. For non-randomized studies, we evaluated their risk of bias by the Risk of Bias in Non-randomized Studies of Intervention (ROBINS-I) assessment tool [14]. Finally, we determined effective intervention programs in African school settings from these results.

## Results

### Results of literature search

**Fig 1:**
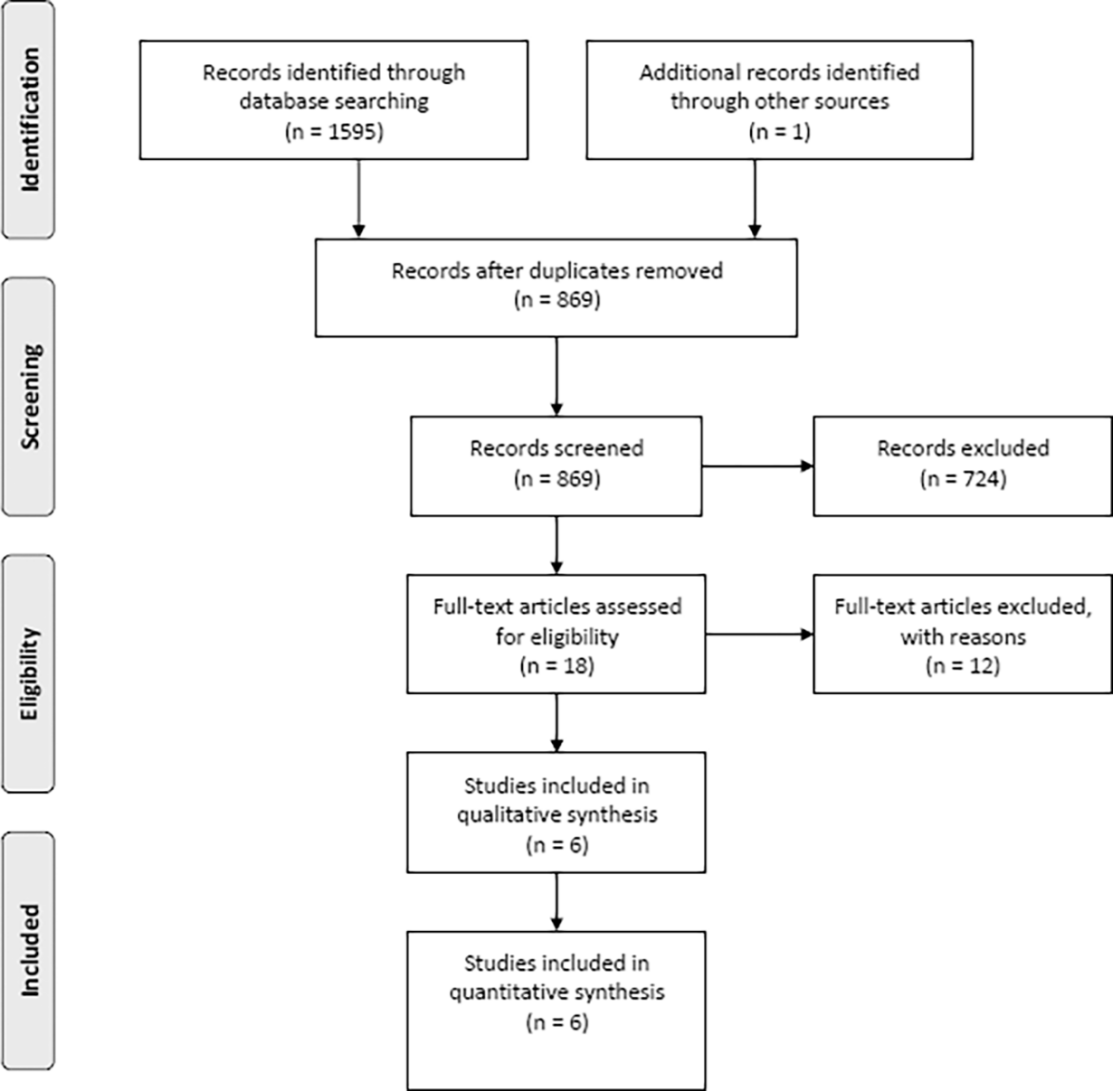
Flow chart of the review.

**Fig 2:**
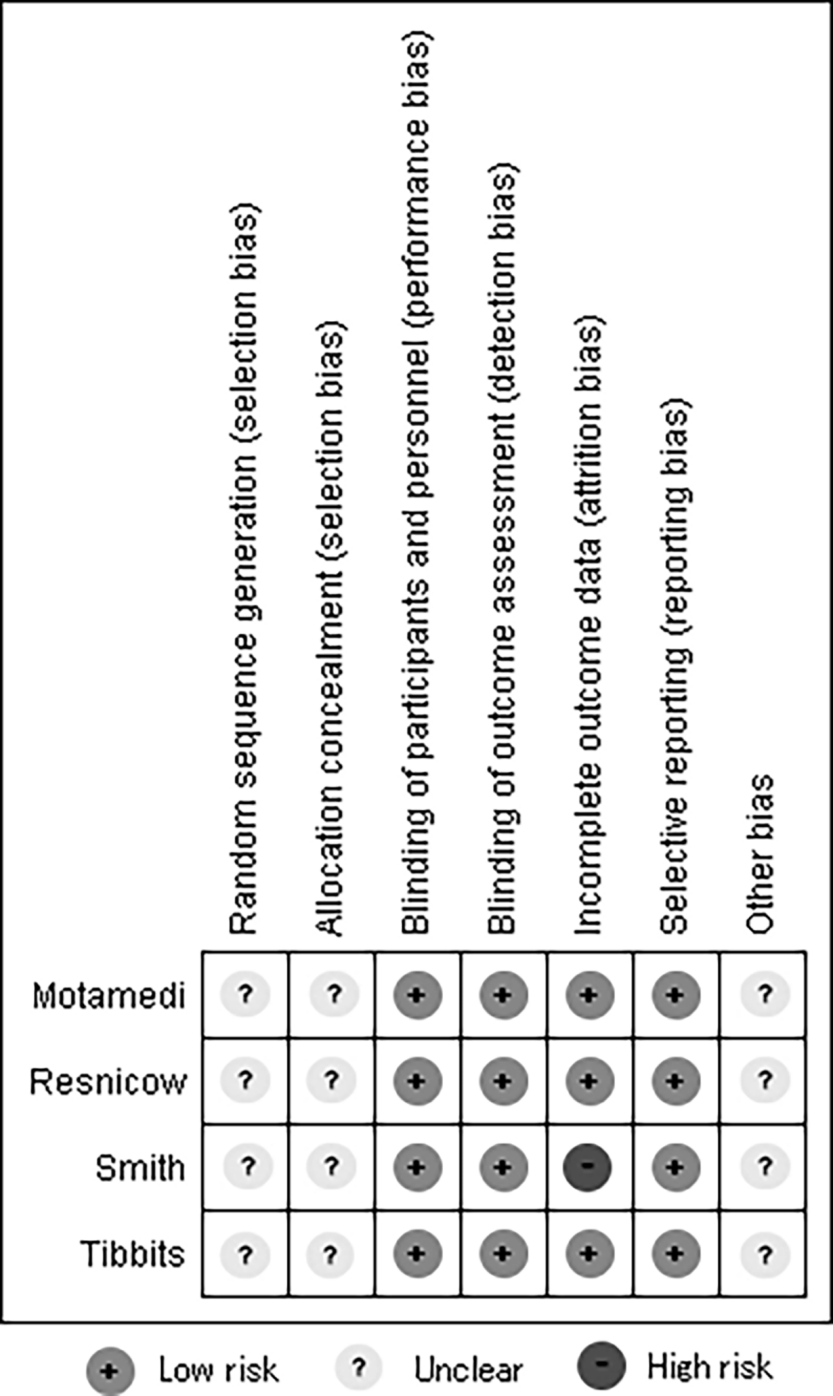
Risk of bias for RCT studies.

**Fig 3:**
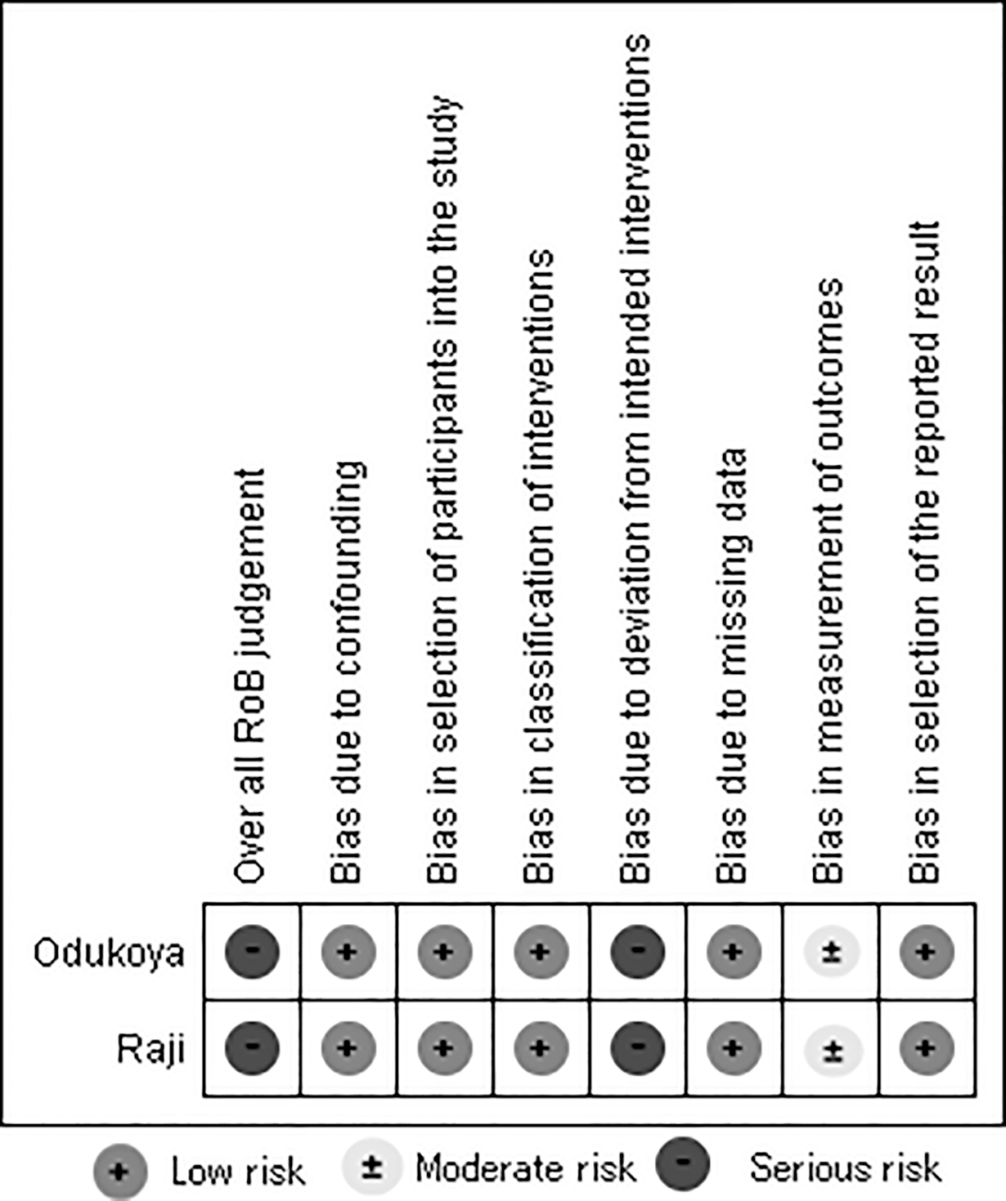
Risk of bias for non-RCT studies.

The PubMed search yielded 549 articles. Searches of Web of Science, ERIC, PsycINFO, Popline, CINAHL, and CENTRAL yielded 173, 13, 206, 51, 19 and 25 articles, respectively. SCOPUS limited the length of search formula. Therefore, we divided the section of the formula concerning country name into three parts and conducted three separate searches. These searches yielded 220, 184, and 155 articles, respectively. We found one article through a hand search. The most common reasons for exclusion were that studies were not prevention studies at schools (e.g., simple smoking prevalence surveys). Six articles [15–20] were included in the review. Fig 1 shows our flow chart of the review to selected articles. Table 1 shows the outline of the included articles. Figs 2 and 3 were created using Review Manager 5.3 and show the evaluation of their risk of bias.

**Table 1:** Outline of included articles.

RCTs study								
Ref.	Publish year	Authors	Country	Years of data	Targeted and control population	Intervention	Outcomes	Mainfindings
15	2008	Resnicow et al.	South Africa	2004–2008	1751, 1529, and 1404 of Grade 8 learners for control, LST and HM	Life skill training (LST) or harm minimization (HM) curriculum. Each intervention program comprised 8 units for both grade 8 and 9.	Smoking rate in last 30 days, knowledge and attitude towards smoking	No significant difference was found in the rate of 30-days smoking in LST group, HM group and the control group after 1 and 2 years follow-up nor in knowledge, attitudes, or skills, to refuse smoking among the three groups.
16	2008	Smith et al.	South Africa	2003–2005	901 and 1275 for intervention and control of Grade 8 learners	Modified Health Wise (HW) program, which was developed in US. The program consists of 12 lessons in grade 8, followed by 6 booster lessons in grade 9.	Smoking rate in lifetime and past 4 weeks	HW girls were significantly less likely to initiate smoking, or to have smoked in the past month, compared to control girls. However, there were no treatment effects among baseline non-smoking boys on these two outcomes. Among the full sample (both baseline smokers and nonsmokers), increases in past-month and heavy smoking were larger for the control group. Heavy smoking was lower among the HW subsample who had not smoked prior to the beginning of the program.
17	2011	Tibbits et al.	South Africa	2004–2005	53% and 47% of 4040 for intervention and control of Grade 8 learners	The intervention was based on HW curriculum. These lessons were provided 18 times from 8th grade to 9th grade.	Lifetime and previous 30 day polydrug use rate, including tobacco	There were not significant gender, cohort, or treatment main effects for lifetime polydrug use. Results of the main effect models for past 30 days polydrug use showed there was no significant difference in analysis of all participants. However,among non users, there was a significant effect in cohort by treatment interaction (β = 0.12, SE = 0.06, *p* < 0.05).
18	2016	Motamedi et al.	South Africa	2004–2008	37%, 63% for intervention and control, respectively of 5610 of 8th and 9th grade	Modified HW program. The program consists of 12 lessons in 8th grade and 6 lessons in 9th grade.	Lifetime smoking rate	Among baseline non-smokers, HW’s effect on preventing cigarette use by the start of 10th grade was moderated in girls (OR = .64, *p* = .02). The likelihood of initiating cigarette use was reduced in girls with the intervention, (OR = .73, *p* = .01) but not boys’ (OR = 1.14, *p* = .35).
Cohort Study								
19	2014	Raji et al.	Nigeria	2012	114 and 114 for intervention and control of senior high school students	The intervention consisted of 2 peer led health education sessions. Each session lasted about 60 minutes, and was repeated 4 weeks after the first health education intervention.	Smoking rate in last 30 days, knowledge and attitude towards smoking	No significant difference was found in the rate of 30-days smoking in intervention group and the control group.The mean knowledge score of respondents in the study group significantly increased from 61.24 before the intervention to 92.31 after the intervention (*p <* 0.01). Attitudes towards smoking also changed. For example, 71.9% of respondents in the study group at baseline felt that cigarettes should not be sold to people less than 18 years old, this increased to 91.7% post-intervention (*p <* 0.01).
20	2014	Odukoya et al.	Nigeria	2009–2010	478 and 495 for intervention and control of junior and senior secondary schools students	The intervention was based on the anti-smoking awareness program which was developed on the Health Belief Model of behavioral change. This program consisted of two health talks about the effects of smoking on health for one hour, providing information leaflets, and putting posters within school.	Smoking rate in last 30 days, knowledge and attitude towards smoking	No significant difference was found in the rate of 30-days smoking in intervention group and the control group. Students in the intervention group had significantly higher mean knowledge scores after intervention program (p < 0.001). The mean score of attitudes towards smoking was also significantly higher in the intervention group (p < 0.001).

### Risk of bias

Selection bias of four RCTs survey [15–18] was not evaluated enough because of lack of information. Performance and detection bias of four RCT was equally judged a low risk among four RCTs studies since the intervention group and control group were separated by school. Percentage attrition of Resnicow et al.[15], Smith et al.[16], Tibbits et al.[17], Motamedi et al [18] were 11%, 38%, 10% and 10%, respectively. All studies measured the last outcome two years or less from baseline. We judged Smith et al. to have a high risk of attrition bias. The other three studies were judged as having low risk of attrition bias since the percentage of attrition was small and the reason for attrition was mainly considered to be natural decrease because of dropping out or repeating a year. Reporting bias of four RCTs surveys was also judged to be low risk since these studies were not related to commercial activities.

Bias due to confounding of two cohort studies was judged a low risk because of very simple surveys at schools. Bias in selection of two cohort surveys was judged a low risk since selection of participants into the survey did not base on participant characteristics. Bias in classification of two cohort surveys was judged a low risk since intervention groups cleary defined. Bias due to deviation of two cohort surveys was equally judged a serious risk since it is considered a lot of other factors such as children’s life style or human relationship affect the results. Bias due to missing data of two cohort surveys was judged a low risk since the percentage attrition of Raji et al.[19], Odukoya et al[20] were 4.4%, 2.5%, respectively and very low. Bias in measurement of outcomes was judged moderate risk in two cohort surveys since the outcome would be partially influenced by children’s interest of tobacco issue. Bias of selection of reported result was judge a low risk in two cohort surveys since these studies were considered related to any intention of authors.

The study design was very similar in every study. Therefore, evaluation of risk of bias was similar for all studies.

### Characteristics of included studies

Four studies [15–17, 20] were conducted in South Africa and the other two [19,20] were conducted in Nigeria. One study [19] targeted high school students, four studies [15–18] targeted junior high school students, and one study [20] targeted both junior high and high school students. Four intervention programs [15–18] were systematically incorporated into annual curriculums, and targeted 8^th^- to 9^th^-grade learners. The other two interventions [19,20] were temporary programs. Classroom lecture education using a textbook was the standard education style in the six studies. The intervention intensity varied from 2 to 18 sessions. The intervention programs, which contained a higher amount of sessions, also included other health related issues. Thus, the intensity of intervention programs against smoking was considered relatively similar. All intervention programs were based on the hypothesis that providing knowledge or social skills against smoking were helpful, in other words, skills-based programs. The Health Wise (HW) program was one of the skills-based programs. This was modified and used as intervention program in three studies [16,17,18]. The HW program is a school-based prevention program developed to reduce substance use and risky sexual behavior in South Africa [21]. One study [15] used two types of intervention programs: one skills-based program and a second program based on the hypothesis that elimination of smoking entirely was not attainable, with the focus placed instead on the reduction of adverse physical, psychological, and social consequences of heavy use.

All six studies utilized smoking or polydrug use rates before and after program implementation as outcome measures. There were no significant differences between intervention and control groups in three studies [15,19,20], and the effectiveness of the other three studies [16–18] was only partial. One article [16] showed that girls of the intervention group were significantly less likely to have initiated smoking and to have smoked in the past month. Another study [17] showed that non-polydrug users at baseline in the intervention group showed a significantly lower onset of frequent polydrug use, when compared to the control group. Although this study did not evaluate the effects on smoking independently, the findings suggest that the program may have been somewhat effective in preventing substance use overall, including smoking, among adolescents in South Africa. The other study [18] showed that non-smoking girls of the intervention group were significantly less likely to have initiated smoking.

Three studies [15,19,20] also used change of knowledge and/or attitudes towards smoking as outcome measures. Two [19,20] of these studies showed significant differences between intervention and control groups. The other [15] did not show any significant difference.

In total, only five studies [16–20] reported positive results of programs focused on smoking prevention. All of these studies could only demonstrate partial effectiveness.

In the study by Resnicow et al. [15], 36 public schools were randomly recruited from two provinces in South Africa. The total number of valid participants was 4684, and the 36 schools were randomly assigned into three groups: control group, Life Skills Training (LST) group, and Harm Minimization (HM) group. The LST program, which was one of the skills-based programs developed in the U.S., has been shown in several randomized trials to reduce tobacco and other substance use [22–33] and is a common program for tobacco intervention utilized worldwide [34]. The LST program included the following elements: training in social skills, training in problem solving, enhancement of self-esteem, correction of overestimations of tobacco consumption, preparation for facing puberty-related physical changes without stress, and information on the effects of tobacco consumption on health. On the other hand, the concept of the HM program is that eliminating cigarette and other drug use entirely is neither philosophically tenable, nor practically attainable [35,36]. The focus of HM is on reducing adverse physical, psychological, and social consequence of use, particularly heavy use. Each intervention program comprised eight units each of grade 8 and 9. The two intervention program was designed to be taught by life orientation (LO) teachers. LO is a separate mandatory topic in South Africa which includes student outcomes for health behaviors and social skills development. Effectiveness was measured by smoking rate in the past 30 days, knowledge/attitudes toward smoking, and refusal skills at the beginning of eighth grade as baseline, and later in eighth and ninth grade as the time points following intervention. The rate of smoking in the past 30 days in the control group at baseline increased at 1 and 2 year follow-ups (18% vs. 21% vs. 24%). The corresponding rates in the LST group were 17% vs. 20% vs. 20%, respectively. The corresponding rates in the HM group were 17% vs. 18% vs. 20%. No significant difference at 1 and 2-year follow-ups was noted among the three groups. Overall, knowledge/attitudes towards smoking and refusal skills demonstrated no significant differences among the three groups. It was mentioned that the HM program appeared to be more effective for black African students, whereas the LST program appeared to be more effective in reducing the 30-day smoking rate in other demographics.

Smith et al. [16] randomly selected four junior high schools as an intervention group, and 15 junior high schools as a control group, in the Mitchell’s Plain area of South Africa. Participants were a total of 2383 8^th^-grade students. The intervention was based on the HW curriculum, and was designed based on the premise that increasing basic life skills, knowledge of the risks associated with substance use/sexual behavior, and enhancing the skills needed to resist substance use/sex is necessary. It was also considered that promoting healthy free time experiences would decrease substance use and sexual behaviors among youth [37–39]. The program consisted of 12 lessons in 8th grade and 6 booster lessons in 9th grade. Each lesson took two or three class periods to deliver. Lessons covered topics included in most social-emotional skills programs (e.g., anxiety/anger management, decision making, self-awareness), and also targeted the positive use of free time (e.g., beating boredom, overcoming leisure constraints, leisure motivation). These lessons were complemented by specific lessons on attitudes, knowledge, and skills surrounding sexual risk and substance use and sexual risk, including tobacco use. The lessons were provided in either English or Afrikaans. Effectiveness was measured by lifetime smoking rate and frequency of use in the past four weeks. Data were collected at the beginning and end of 8th grade. The results showed HW girls were significantly less likely to have initiated smoking, or to have smoked in the past 30 days, as compared to control group girls. However, there were no treatment effects among baseline non-smoking boys on these two outcomes. Among the full sample (both baseline smokers and non-smokers), increases in past 30 days and heavy smoking were also larger for the control group. Heavy smoking was also lower among the HW group who had not smoked prior to the beginning of the program.

Tibbits et al. [17] randomly selected four schools as an intervention group and five schools as a control group in Cape Town, South Africa. The total participants were 4040 8^th^-grade students. The intervention was based on the HW curriculum. The program consisted of 18 lessons from 8th to 9th grade. The effectiveness was measured by the lifetime and previous 30-day polydrug use rate, including tobacco. Data were collected at the beginning and end of 8th grade. The results showed no significant effects of the HW program relating to gender, cohort, or treatment for lifetime polydrug use. Females of the intervention group had a smaller increase (32%) in substance use than females in the control group (36%). However, the results of the main effect models for polydrug use in the past 30 days showed no significant difference in analysis of all participants. On the other hand, the rate of polydrug use in the HW program group was significantly lower than the control group among non-users (β = 0.12, SE = 0.06, p < 0.05).

Motamedi et al. [18] randomly selected four high schools as an intervention group and 15 schools as control group in the Mitchell’s Plain area of South Africa. A total of 5610 high school students participated. The intervention was based on HW curriculum. The program consisted of 12 lessons (each approximately two to three class periods long) in 8th grade and six lessons in 9th grade. Lessons were provided in either English or Afrikaans. Effectiveness was measured by lifetime smoking rate. Data were collected through self-report surveys for youth prior to the start of the intervention, in the first two months of the beginning of 8th grade (pre-intervention), and at the start of 10th grade (follow-up). The results showed that HW’s effect on preventing cigarette use by the start of 10th grade was moderated among females who were non-smokers at baseline [odds ratio (OR) = .64, p = .02. The likelihood of initiating cigarette use was reduced by the intervention in females (OR = .73, p = .01), but not in males (OR = 1.14, p = .35).

Raji et al. [19] recruited 114 students each for study and control groups in senior secondary schools in Sokoto metropolis, Nigeria. The intervention consisted of two peer-led health education sessions. Each session consisted of a didactic lecture, showing an 18-minute video clip, and interactive discussion. Each session lasted about 60 minutes and was repeated four weeks after the first health education intervention. Effectiveness was measured by a 44-item, self-administered questionnaire, modified from the core questions of the Global Youth Tobacco Survey. The questionnaire consisted of four sections; specifically, information regarding demographic characteristics, knowledge about smoking, attitudes toward smoking, and behavior of respondents related to smoking. Data were collected before and three months after the intervention program. Results showed the mean knowledge score of respondents significantly increased, from 61.24 prior to the intervention, to 92.31 after the intervention (*p* < 0.001). Attitudes towards smoking also changed significantly. For example, 71.9% of respondents in the study group at baseline felt that cigarettes should not be sold to people less than 18 years old, and this increased to 91.7% post-intervention (p < 0.001). After the intervention, the number of students who had smoked in the last 30 days decreased by 0.6% in the intervention group (7.9% vs. 7.3%), whereas this number increased by 0.1% in the control group (8.8% vs. 8.9%). These differences were not statistically significant.

Odukoya et al. [20] randomly selected three schools for the intervention group and three for the control group from Lagos State in Nigeria. The minimum sample size for the study was calculated, and one or two classes were randomly selected from each of the five grades. A total of 511 and 520 students were in the selected classes in the intervention and control groups, respectively. The intervention was based on the anti-smoking awareness program, which was developed based on the Health Belief Model (HBM) of behavioral change. The HBM is a psychological model that addresses individuals’ perceptions of threat posed by health problems, the benefits of avoiding the threat, and factors influencing the decision to act. This anti-smoking program consisted of the following components; two talks about the effects of cigarette smoking on health for one hour, providing information leaflets, and putting posters up within the school environment. For research, assistants were given a one-day training organized by the research team. The training covered all aspects of study including pre-testing, data collection and the intervention. Effectiveness was measured by an instrument created by the authors, which consisted of 16 knowledge, seven attitude, and seven practice items. The data were collected before and three months after the intervention program. The results showed students in the intervention group had significantly higher mean knowledge scores after the intervention program (*p* < 0.001) compared to the control group. The mean score of attitudes toward smoking was also significantly higher in the intervention group (*p* < 0.001). After the intervention, the number of students who had smoked in the last 30 days decreased by 1.0% in the intervention group (4.0% vs. 3.0%), whereas this number increased by 0.5% in the control group (3.5% vs. 3.5%). However, these differences were not statistically significant.

## Discussion

These six selected studies suggest that school-based interventions might have some positive effects on improving knowledge level of smoking and attitudes towards smoking, and partially prevent the increase of smoking prevalence among secondary school students in Africa. However, we could not find robust evidence that school-based interventions decreased smoking prevalence among school children in Africa. Identical to our analysis, a Cochrane review reported that school-based interventions in other areas of the world could not detect a statistically significant decrease in the number of current smokers over time [10].

We must consider why these six studies could not show strong evidence that school-based interventions were effective in reducing smoking prevalence in individual students or populations at large, although selected studies showing improved knowledge and attitudes about smoking and/or increased smoking prevalence. As a reason, the limited quality and inconsistent outcome measurements can be considered as follows.

First, the purposes and outcome measurements were inconsistent for each intervention. In the six selected studies, students targeted for intervention were mixed, composed of non-smokers, non-smokers who were likely to initiate smoking, and current smokers. Therefore, interventions attempted to play a role in preventing the initiation of smoking, quitting smoking, and/or reducing tobacco or other drug dangers. Such multiple focuses may weaken the effects for each group of target students. Specific analysis for each type of student may be required to increase the power of interventions.

Second, it is possible the training for presenters was not appropriate or not sufficient. Only Resnicow et al.[15] and Odukoya et al. [20] referred to qualification of the presenters. Well trained presenters might affect the children’s smoking behavior.

Third, the interventions did not focus on the modification of various factors that induce smoking behaviors in adolescents. The selected studies showed the different impacts of intervention by gender [16,20]. Previous literature in Africa also showed different effects by age, other substance use, socio-economic status, mental status, physical activities, and rural-urban location among African countries [40–44]. One cohort, conducted by DeVries et al. in six European countries [45] demonstrated that adolescents’ smoking onset was influenced by parental behavior and choice of friends with similar smoking behavior. If an intervention can somehow modify various factors that induce smoking among students, actual smoking behavior could be more easily affected. A brief review of school-based prevention approaches targeting individual-level etiologic factors demonstrated some effectiveness of interventions [46].

Fourth, a comprehensive approach may be needed to enhance the effectiveness of interventions. All of the selected six studies created intervention programs only from the perspective of skills-based health education within the FRESH framework. It also may be effective to combine with other approaches including health-related school policy statements, education for community people, and increases in tobacco tax. The Health Promoting Schools Framework provided by the WHO suggests the importance of collaboration with parents and local communities to develop effective intervention programs in schools [47]. The cultural world of adolescents (internet, teen idols, and media) are also important components of programs delivered through the Internet.

Currently, the global trend of smoking is in decline. In light of this, tobacco companies have begun to expand their markets in low- and middle-income countries, capitalizing on economic growth, changing social norms, and population demographics. Africa has lower rates of tobacco taxation, weaker smoke-free policies, and less stringent tobacco advertising restrictions in comparison to high-income countries. In countries such as Africa, school-based anti-smoking intervention programs have many strong points. Since the enrolment rate of primary school increased from 75.2% in 1990 to 99.2% in 2013 [48] in Africa, intervention programs can now, theoretically, be provided to nearly all children. Additionally, these programs are cost-effective and sustainable. This study showed that school-based interventions may be effective in improving knowledge and attitudes about smoking, and partially effective in preventing increased smoking prevalence among secondary school students in Africa. However, we could not find robust evidence that school-based interventions decreased smoking prevalence. Multi-model, school-based smoking prevention programs, and studies that aim to change the school environment and state policies, with wider initiatives within and beyond the school, including programs for parents, schools, and communities, are needed in Africa. Commonly, tobacco use is a gateway to the use of other substances in later life stages. Comprehensive and multi-disciplinary approaches are needed to provide powerful evidence of the effectiveness of such programs.

### Limitations

The limitations of this review are that only a few papers were selected, and those included were only carried out in South Africa and Nigeria. Africa contains extreme diversity in religion, race, and school systems. Therefore, this review may not represent the results for school-based programs on preventing tobacco use in the whole of Africa. Furthermore, there were no “pure prevention cohort” but groups of mixed never-smokers and smokers. Even with these limitations, this study clarified weak points of existing studies, and provided future directions for study design within this field.

### Conclusions

There were four RCTs studies and two cohort studies of school-based tobacco prevention program in African countries from 2000 to 2016. The all of RCTs studies showed no significant change of smoking-rate by the intervention. The effectiveness of intervention was observed only in some sub-group. The cohort studies showed school-based interventions may be effective in improving knowledge and attitudes about smoking. However, they reported no significant change of smoking-rate by the intervention.

## Supporting information

S1 File Prisma doc. This is the PRISMA 2009 checklist.
(DOC)

## References

[1] American Cancer Society. Cancer facts and figures. 2005. Available from: http://our.cancer.org/downloads/STT/CAFF2005f4PWSecured.pdf.

[2] U.S. Department of Health and Human Services. The health consequences of smoking: a report of the surgeon general. U.S. Department of Health and Human Services, Centers for Disease Control and Prevention, Office on Smoking and Health; 2004.

[3] Eriksen M, Mackay J, Ross H. The Tobacco Atlas, Fourth Edition Atlanta Georgia: American Cancer Society, Inc.; 2012 Available from: http://tobaccoatlas.org/uploads/Images/PDFs/Tobacco_Atlas_2ndPrint.pdf.

[4] World Health Organization. Tobacco key facts. 2009. Available from: http://www.who.int/topics/tobacco/facts/en/index.html.

[5] World Health Organization. The MMPOWER package: WHO report on the global tobacco epidemic. Geneva: World Health Organization; 2008 p.1–19.

[6] The World Bank. Smoking prevalence, males; Smoking prevalence, females. Available from: http://data.worldbank.org/indicator/SH.PRV.SMOK.MA, and http://data.worldbank.org/indicator/SH.PRV.SMOK.FE

[7] Lopeze AD, Collishaw NE, Piha T. A descriptive model of the cigarette epidemic in developed countries. Tob Control. 1994; 3: 242–247.

[8] National Cancer Institute and World Health Organization. The economics of tobacco and tobacco control. National Cancer Institute Tobacco Control Monograph 21. Bethesda: National Institutes of Health; and Geneva: World Health Organization; 2016 Available from: https://cancercontrol.cancer.gov/brp/tcrb/monographs/21/docs/m21_complete.pdf

[9] UNESCO. FRESH: a comprehensive school health approach to achieve EFA. Paris: UNESCO; 2002 Available from: http://unesdoc.unesco.org/images/0012/001255/125537e.pdf

[10] Thomas RE, McLellan J, Perera R. Effectiveness of school-based smoking prevention curricula: systematic review and meta-analysis. BMJ Open. 2015; 5(3): e006976 doi: 10.1136/bmjopen-2014-006976 25757946 10.1136/bmjopen-2014-006976 PMC4360839

[11] Skara S, Sussman S. A review of 25 long-term adolescent tobacco and other drug use prevention program evaluations. Prev Med. 2003; 37: 451–474. 14572430 10.1016/s0091-7435(03)00166-x

[12] Wiehe SE, Garrison MM, Christakis DA, Ebel BE, Rivara FP. A systematic review of school-based smoking prevention trials with long-term follow-up. J Adolesc Health. 2005; 36: 162–169. doi: 10.1016/j.jadohealth.2004.12.003 15737770 10.1016/j.jadohealth.2004.12.003

[13] The Cochrane Collaboration’s tool for assessing risk of bias. Available from: http://handbook.cochrane.org/chapter_8/8_5_the_cochrane_collaborations_tool_for_assessing_risk_of_bias.htm

[14] ROBINS-I tool. Available from: https://sites.google.com/site/riskofbiastool/welcome/home

[15] Resnicow K, Reddy SP, James S, Omardien RG, Kambaran NS, Langer HG, et al Comparison of two school-based smoking prevention programs among South African high school students results of a randomized trial. Ann Behav Med. 2008; 36: 231–243. doi: 10.1007/s12160-008-9072-5 19067098 10.1007/s12160-008-9072-5

[16] Smith EA, Palen LA, Caldwell LL, Flisher AJ, Graham JW, Mathews C, et al Substance use and sexual risk prevention in Cape Town, South Africa: an evaluation of the HealthWise program. Prev Sci. 2008; 9: 311–321. doi: 10.1007/s11121-008-0103-z 18836890 10.1007/s11121-008-0103-z

[17] Tibbits MK, Smith EA, Caldwell LL, Flisher AJ. Impact of HealthWise South Africa on polydrug use and high-risk sexual behavior. Health Educ Res. 2011; 26: 653–663. doi: 10.1093/her/cyr024 21511818 10.1093/her/cyr024 PMC3139488

[18] Motamedi M, Caldwell L, Wegner L, Smith E, Jones D. Girls just want to know where to have fun: preventing substance use initiation in an under-resourced community in South Africa through HealthWise. Prev Sci. 2016; 17: 700–709. doi: 10.1007/s11121-016-0654-3 27129478 10.1007/s11121-016-0654-3 PMC4969046

[19] Raji M, Abubakar I, Oche M, Kaoje A, Isah B. Using peer led health education intervention to improve in-school adolescents’ cigarette smoking related knowledge, attitude and behaviour in a north west Nigeria state. Health Sci J. 2014; 8: 485–494.

[20] Odukoya OO, Odeyemi KA, Oyeyemi AS, Upadhyay RP. The effect of a short anti-smoking awareness programme on the knowledge, attitude and practice of cigarette smoking among secondary school students in Lagos state, Nigeria. Niger Postgrad Med J. 2014; 21: 128–135. 25126866

[21] Caldwell LL, Smith EA, Flisher AJ, Wegner L, Vergnani T, Mathews C, et al Health Wise South Africa: development of a life skills curriculum for young adults. World Leis. 2004; 3: 4–17.

[22] Botvin G, Eng A. The efficacy of a multicomponent approach to the prevention of cigarette smoking. Prev Med. 1982; 11: 199–211. 7088907 10.1016/0091-7435(82)90018-4

[23] Botvin G, Baker E, Renick N, Filazzola A, Botvin E. A cognitive-behavioral approach to substance abuse prevention. Addict Behav. 1984; 9: 137–147. 6611026 10.1016/0306-4603(84)90051-0

[24] Botvin G, Dusenbury L, Baker E, James-Ortiz S, Kerner J. A skills training approach to smoking prevention among Hispanic youth. J Behav Med. 1989; 12: 279–295. 2634104 10.1007/BF00844872

[25] Botvin GJ, Baker E, Dunsenbury L, Tortu S, Botvin E. Preventing adolescent drug abuse through a multimodal cognitive-behavioral approach: results of a three year study. J Consult Clin Psychol. 1990; 58: 437–446. 2212181 10.1037//0022-006x.58.4.437

[26] Botvin G, Baker E, Dusenbury L, Botvin E, Diaz T. Long-term follow-up results of a randomized drug use prevention trial in a white middle-class population. JAMA. 1995; 273: 1106–1112. 7707598

[27] Botvin G, Epstein J, Schinke S, Diaz T. Predictors of cigarette smoking among inner-city minority youth. J Dev Behav Pediatr. 1994; 15: 67–73. 8034769

[28] Botvin G, Dusenbury L. Smoking prevention among urban minority youth: Assessing effects on outcome and mediating variables. Health Psychol. 1992; 11: 290–299. 1425546 10.1037//0278-6133.11.5.290

[29] Botvin GJ, Schinke SP, Epstein JA, Diaz T, Botvin EM. Effectiveness of culturally focused and generic skills training approaches to alcohol and drug abuse prevention among minority adolescents: Two-year follow-up results. Psychol Addict Behav. 1995; 9: 183–194.

[30] Botvin GJ, Schinke SP, Epstein JA, Diaz T. Effectiveness of culturally focused and generic skills training approaches to alcohol and drug abuse prevention among minority youths. Psychol Addict Behav. 1994; 8: 116–127.

[31] Botvin GJ, Griffin KW, Diaz T, Miller N, Ifill-Williams M. Smoking initiation and escalation in early adolescent girls: one year follow-up of a school-based prevention intervention for minority youth. J Am Med Women’s Assoc. 1999; 54: 139–143,152. 10441920

[32] Botvin GJ, Griffin KW, Diaz T, Scheier LM, Williams C, Epstein JA. Preventing illicit drug use in adolescents: long-term follow-up data from a randomized control trial of a school population. Addict Behav. 2000; 25: 769–774. 11023017 10.1016/s0306-4603(99)00050-7

[33] Botvin GJ. Preventing drug abuse in schools: social and competence enhancement approaches targeting individual-level etiologic factors. Addict Behav. 2000; 25: 887–897. 11125777 10.1016/s0306-4603(00)00119-2

[34] Seal N. Preventing tobacco and drug use among Thai high school students through life skills training. Nurs Health Sci. 2006; 8: 164–168. doi: 10.1111/j.1442-2018.2006.00275.x 16911176 10.1111/j.1442-2018.2006.00275.x

[35] Resnicow K, Smith M, Harrison L, Drucker E. Correlates of occasional tobacco and marijuana use: Are teens harm reducing? Addict Behav. 1999; 24: 251–266. 10336106 10.1016/s0306-4603(98)00059-8

[36] Hamilton G, Cross D, Resnicow K. Occasional cigarette smokers: Cue for harm reduction smoking education. Addict Res. 2000; 8: 419–437.

[37] Caldwell LL, Smith EA, Flisher AJ, Mathews C. HealthWise South Africa: development of a life skills curriculum for young adults. World Leis 2004; 3: 4–17.

[38] Smith EA, Palen L-A, Caldwell LL, Flisher AJ, Graham JW, Mathews C, et al Substance use and sexual risk prevention in Cape Town, South Africa: an evaluation of the HealthWise program. Prev Sci. 2008; 9: 311–21. doi: 10.1007/s11121-008-0103-z 18836890 10.1007/s11121-008-0103-z

[39] Wegner L, Flisher AJ, Caldwell LL, Vergnani T, Smith EA. Healthwise South Africa: cultural adaptation of a school-based risk prevention programme. Health Educ Res. 2008; 23: 1085–1096. doi: 10.1093/her/cym064 17956882 10.1093/her/cym064 PMC2721676

[40] Peltzer K. Early smoking initiation and associated factors among in-school male and female adolescents in seven African countries. Afr Health Sci. 2011; 11: 320–328. 22275919 PMC3261015

[41] Brook DW, Rubenstone E, Zhang C, Morojele NK, Brook JS. Environmental stressors, low well-being, smoking, and alcohol use among South African adolescents. Soc Sci Med. 2011; 72: 1447–1453. doi: 10.1016/j.socscimed.2011.02.041 21492977 10.1016/j.socscimed.2011.02.041 PMC3090534

[42] Townsend L, Flisher AJ, Gilreath T, King G. A systematic literature review of tobacco use among adults 15 years and older in sub-Saharan Africa. Drug Alcohol Depend. 2006; 84: 14–27. doi: 10.1016/j.drugalcdep.2005.12.008 16442750 10.1016/j.drugalcdep.2005.12.008

[43] Brathwaite R, Addo J, Smeeth L, Lock K. A systematic review of tobacco smoking prevalence and description of tobacco control strategies in Sub-Saharan African countries; 2007 to 2014. PloS one. 2015; 10: e0132401 doi: 10.1371/journal.pone.0132401 26162085 10.1371/journal.pone.0132401 PMC4498629

[44] Achia TN. Tobacco use and mass media utilization in sub-Saharan Africa. PloS one. 2015; 10: e0117219 doi: 10.1371/journal.pone.0117219 25706131 10.1371/journal.pone.0117219 PMC4338150

[45] De Vries H, Candel M, Engels R, Mercken L. Challenges to the peer influence paradigm: results for 12–13 year olds from six European countries from the European Smoking Prevention Framework Approach study. Tob Control. 2006; 15: 83–89. doi: 10.1136/tc.2003.007237 16565454 10.1136/tc.2003.007237 PMC2563573

[46] Botvin GJ. Preventing drug abuse in schools: Social and competence enhancement approaches targeting individual-level etiologic factors. Addict Behav. 2000; 25: 887–897. 11125777 10.1016/s0306-4603(00)00119-2

[47] World Health Organization. Health Promoting Schools Framework. Geneva: World Health Organization; 2009 Available from: http://www.wpro.who.int/health_promotion/documents/docs/HPS_framework_for_action.pdf?ua=1

[48] UNESCO. Gross enrolment ratio by level of education. Paris: Unesco; n.d. Available from: http://data.uis.unesco.org/?queryid=142#

